# Inspiratory Muscle Training in Patients With Heart Failure With Preserved Ejection Fraction: A Meta-Analysis

**DOI:** 10.7759/cureus.12260

**Published:** 2020-12-24

**Authors:** Nischit Baral, Hameem U Changezi, Mahin R Khan, Govinda Adhikari, Prakash Adhikari, Hafiz Muhammad Waqas Khan, Abhushan Poudyal, Basel Abdelazeem, Shashi Sigdel, Andrew Champine

**Affiliations:** 1 Internal Medicine, McLaren Flint/Michigan State University (MSU), Flint, USA; 2 Cardiovascular Medicine, McLaren Flint/Michigan State University (MSU), Flint, USA; 3 Cardiology, McLaren Flint/Michigan State University (MSU), Flint, USA; 4 Internal Medicine, McLaren Flint, Flint, USA; 5 Internal Medicine, Piedmont Athens Regional Medical Center, Athens, USA; 6 Cardiovascular Medicine, Cook County Health, Chicago, USA; 7 Internal Medicine, Rochester Regional Health, Rochester, USA; 8 Behavioral Health/Family Medicine/Internal Medicine, McLaren Flint/Michigan State University (MSU), Flint, USA

**Keywords:** inspiratory muscle training, heart failure with preserved ejection fraction, respiratory muscle exercise, diastolic heart failure, peak oxygen consumption, 6 minute walk distance

## Abstract

Objectives

To explore the role of inspiratory muscle training (IMT) in improving cardiorespiratory fitness of stable heart failure with preserved ejection fraction (HFpEF) patients.

Background

There is a paucity of data on the role of IMT in patients with HFpEF. HFpEF is a growing problem in the developed world, especially in the aging population.

Methods

We conducted a systematic literature search for English studies in PubMed, EMBASE, and Cochrane Central Register of Controlled Trials. We searched databases using terms relating to or describing breathing exercise, IMT, and HFpEF. RevMan 5.4 (The Cochrane Collaboration, 2020) was used for data analysis, and two independent investigators performed literature retrieval and data extraction.

Results

We identified three randomized controlled trials (RCTs) and one prospective study on the role of IMT in HFpEF. We calculated the pooled mean difference of peak oxygen consumption (Peak VO_2_) and six-min walk distance (6MWD) between the IMT and standard care (SC) groups. Our meta-analysis showed that compared with SC, IMT could significantly improve peak VO_2_ with a mean difference (MD) of 2.82 ml/kg/min, 95% CI [1.90, 3.74] P < 0.00001 and improve 6MWD with MD of 83.97 meters, 95% CI [59.18, 108.76] P< 0.00001 to improve cardiorespiratory fitness at 12 weeks of IMT and improve peak VO_2_ with MD of 2.18 ml/kg/min, 95% CI [0.38, 3.99] P < 0.00001 at 24 weeks of therapy.

Conclusion

IMT should be further studied as a possible treatment option to improve cardiorespiratory fitness for patients with stable HFpEF.

## Introduction

According to the American Heart Association Heart Disease and Stroke Statistics 2020 update, heart failure affects 6.2 million in the United States alone [1-2]. In the US, more than 650,000 new patients are diagnosed with heart failure and at least half of them are HFpEF [3]. More than 80% of heart failure in females is heart failure with preserved ejection fraction (HFpEF). Almost all of the heart failure patients in their 90s have preserved ejection fraction. The health, as well as economic, impact of HFpEF is immense. One-year mortality for HFpEF ranges up to 29%. While the prevalence of HFpEF seems to be increasing, the prognosis seems to be static [4]. Many trials of pharmacological treatments have shown no promising results in reducing mortality. Moreover, we are lacking beneficial therapies to improve the quality of life in patients with stable HFpEF.

Inspiratory muscle weakness (IMW) is seen in patients with both kinds of chronic heart failure, heart failure with reduced ejection fraction (HFrEF) and HFpEF [5]. In our study, we have shifted our gaze to evaluate inspiratory muscle training for improving quality of life and cardiorespiratory fitness in patients with stable HFpEF. We have used peak oxygen consumption (Peak VO_2_) and six-min walk distance (6MWD) as a marker of cardiorespiratory fitness and quality of life in HFpEF patients [4,6-9].

## Materials and methods

Selection of studies

**Figure 1:**
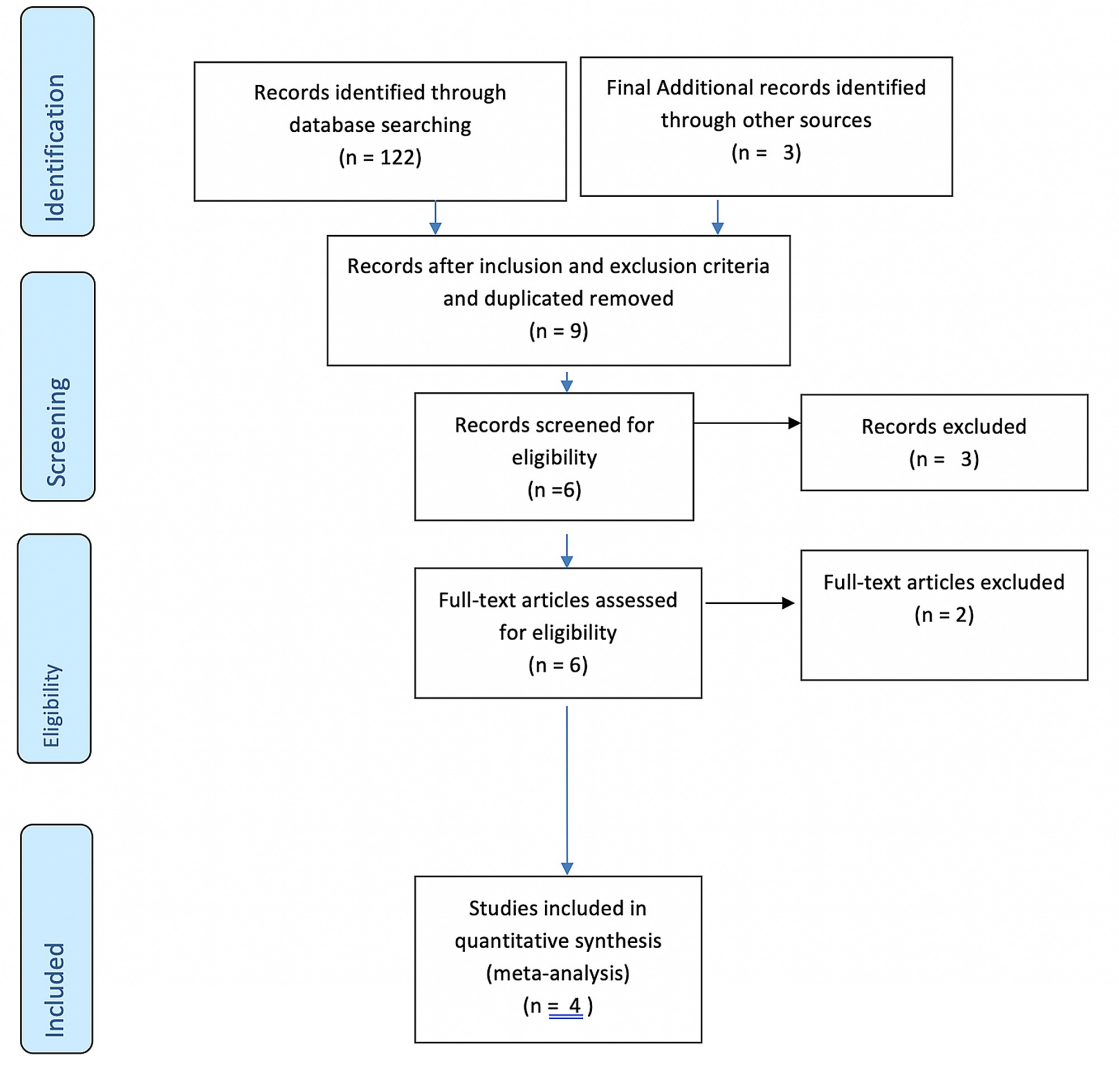
Flow Diagram on Selection of Studies

We searched Medline, Embase, and Cochrane Central Register of Controlled Trials using the terms heart failure with preserved ejection fraction, diastolic heart failure, HFpEF, inspiratory muscle training, respiratory muscle training, and breathing exercise for literature published prior to October 2020. The titles and abstracts of all results were reviewed, and studies were selected for full-text analysis according to the eligibility criteria. From the literature search from Medline and Embase, we could find only find three randomized controlled trials (RCTs) and one prospective study that met all of our inclusion criteria. We also searched Google Scholar and looked at the citations to find any unique articles that met our inclusion criteria. We used the Preferred Reporting Items for Systematic Reviews and Meta-Analyses (PRISMA) flow chart generator for the literature search. The quantitative synthesis was performed using fixed and random effect models in RevMan 5.4 (The Cochrane Collaboration, 2020). Heterogeneity was assessed using the I-squared (I^2^) test. We identified a total of 122 studies after electronic database searching of the Pubmed, Embase, and Cochrane databases and three studies from Google Scholar. Screening of the titles and abstracts of 125 studies was done. We excluded 52 duplicate studies. We excluded 64 studies based on inclusion and exclusion criteria. Finally, we excluded 119 studies and checked six articles for full-text eligibility. We excluded two studies due to multiple interventions in addition to IMT as mentioned in the PRISMA flow diagram in Figure 1. Finally, four studies were selected for meta-analysis.

Objective

To explore the role of Inspiratory muscle training (IMT) in improving the cardiorespiratory fitness of stable HFpEF patients.

Inclusion criteria

Our inclusion criteria included patients with left ventricular ejection fraction (LVEF) ≥ 45-50%, New York Heart Association (NYHA) class I, II, or III, and clinician-diagnosed stable HFpEF patients based on signs, symptoms, and imaging.

Exclusion criteria

Our inclusion criteria included patients with LVEF < 45%, Studies on patients who cannot do a baseline exercise test, with medical conditions like moderate-severe valvular heart disease and acute coronary syndrome or cardiac surgery in the previous three months.

In all the included studies, the patients were instructed to train at home twice daily, for 20 minutes each session during 12 weeks, using a threshold inspiratory muscle trainer at a resistance equal to 25%-30% of their maximal inspiratory pressure (MIP). The MIP was measured weekly in the study done by Palau to monthly in a study done by Kinusaga et al. [10]. Outcomes included pre- and post-intervention 6MWD and peak VO_2_. The 6MWD was defined as the distance covered by the study subjects in six minutes while using detailed instructions to help the subjects to cover the maximum distance during the walk test. Subjects walked at self-graded and self-paced speed.

Peak VO_2_ was calculated as the highest value of VO_^2^ _during the last 20 seconds of exercise and was measured using aerobic exercise testing on a bicycle ergometer with a baseline workload. The workload was increased in a gradual fashion every one min. The patients were monitored using a 12-lead electrocardiogram (ECG) and blood pressure was monitored every two minutes. The gas exchange data and cardiopulmonary variables were averaged every 10 seconds.

Standard care

The standard care (SC) group in our study included the randomized participants who were doing the usual pharmacological and non-pharmacological treatment of HFpEF. The non-pharmacological treatment didn't include IMT. It included usual lifestyle modifications like diet and exercise [8,10-12].

Quality assessment and data extraction

Two authors (N.B. and M.K.) independently performed the study selection, data extraction, and quality assessment. The following data were extracted: study design, participant's characteristics, outcome data of findings, including clinical course and prognosis.

Risk of bias assessment

The Cochrane Collaboration risk of bias tool was used to assess the risk of bias. The quality of included studies was assessed by two authors N.B. and M.K. with the help of the Cochrane Risk of Bias assessment tool. The risk of bias of the included studies was graded on the following aspects as low in random sequence generation, as there was randomization of all participants; medium, as there was no blinding of participants; and low in outcomes and reporting.

## Results

After qualitative and quantitative synthesis, we included four studies in our meta-analysis. We identified three randomized controlled trials (RCTs) and one prospective study on the role of IMT in HFpEF. We calculated the pooled mean difference of peak VO2 and 6MWD between the IMT and SC groups.

Baseline characteristics of the IMT group

The baseline characteristics of the IMT group in our meta-analysis is shown in Table 1. The study by Palau et al. 2014, 2017, and 2019 is homogenous in the NYHA class, training protocol, peak VO_2_, and ECG characteristics. The study participants in the IMT group had to train for 20 minutes twice daily with one supervised session per week [8,11-12]. The participants in a study by Kanagusa et al. trained for 20 minutes once daily, with one supervision per month. The baseline characteristics of standard care group is similar to the IMT group, as all the participants were randomized for matching. The LVEF for all the studies is greater than 50% except for the study by Kinugasa et al. where the LVEF was more than 45%. The IMT session lasted for 20 minutes in each study (Table 1) [8,10-12].

**Table 1:** Baseline Characteristics of the IMT Group *IMT: Inspiratory Muscle Training; †FEV1: Forced Expiratory Volume in 1 Second; ‡NYHA: New York Heart Association; §MIP: Maximum Inspiratory Pressure; ¶LVEF: Left Ventricular Ejection Fraction

Year and Author	NYHA class	Training Protocol for IMT	Mean Age	Mean Peak VO2	Echocardiogram characteristics
2014. Palau et al. [8].	NYHA‡ II/III	20 min twice daily with 1 session supervised per week.	69	10 ml/kg/ml	LVEF¶ ≥50% End diastolic diameter < 60mm, stable HFpEF
2017. Palau et al [11].	NYHA II/III	20 min twice daily with 1 session supervised per week.	70	9.9 ml/kg/ml	LVEF¶ ≥50% End diastolic diameter < 60mm, stable HFpEF
2019. Palau et al [12].	NYHA II/III	20 min twice daily with 1 session supervised per week.	67.8	10.4 ml/kg/ml	LVEF¶ ≥50% End diastolic diameter < 60mm, stable HFpEF
2020 Kinugasa et al [10]	NYHA I-III/IV	20 min once daily with 1 session supervised per month.	NA	14.6 ml/kg/ml	LVEF ≥45%

Outcomes

Pooled Change in PVO_2_ After 12 Weeks IMT

**Figure 2:**
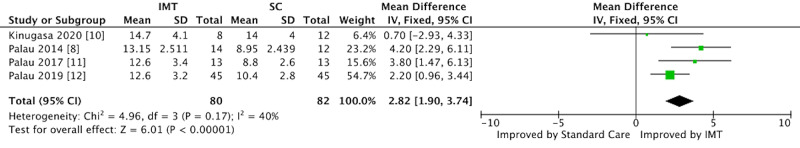
Pooled Change in Peak Oxygen Consumption After 12 Weeks of IMT vs SC IMT: Inspiratory Muscle Training; SC: Standard Care

Our meta-analysis of four studies in patients with HFpEF using a fixed-effect model showed significant improvement in the peak VO_2_ as compared to the SC with MD of 2.82 ml/kg/min, 95% CI [1.90, 3.74] P < 0.00001 (Participants in IMT: 80, Participants in SC: 82, I^2^ = 40%). The results of the study is compared with SC after 12 weeks. The result of this meta-analysis shows that with the regular practice of IMT for 12 weeks in patients with HFpEF, the peak VO_2_ can be improved by 2.82 ml/kg/min as compared with the regular standard therapy for IMT, which includes both pharmacological and non-pharmacological therapies (Figure 2).

Pooled Change in 6MWD After 12 Weeks of IMT

**Figure 3:**
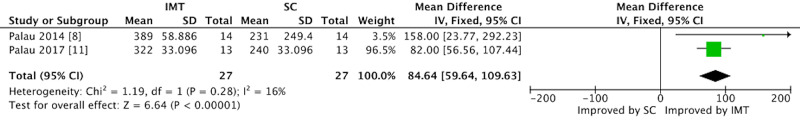
Pooled Change in 6-Minute Walk Distance After 12 Weeks of IMT vs SC IMT: Inspiratory Muscle Training; SC: Standard Care

Our meta-analysis of two studies showed significant improvement in the 6MWD with MD of 83.97 meters, (95% CI [59.18, 108.76] P < 0.00001) to improve cardiorespiratory fitness at 12 weeks of IMT (Participants in IMT: 27, Participants in SC: 27, I^2^=16%). The result of this meta-analysis shows that with the regular practice of IMT for 12 weeks in patients with HFpEF, 6MWD can be improved by 84.64 meters. The participants in IMT could walk 84.64 meters more than the participants in SC in 6 minutes (Figure 3). 

Pooled Change in Peak VO_2 _After 24 Weeks of IMT

**Figure 4:**
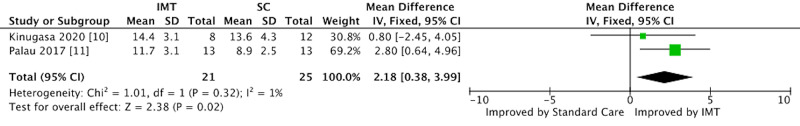
Pooled Change in Peak Oxygen Consumption After 24 Weeks of IMT vs SC IMT: Inspiratory Muscle Training; SC: Standard Care

Our meta-analysis of two studies in patients with HFpEF using the fixed-effect model showed significant improvement in peak VO_2_ compared to SC with MD of 2.18 ml/kg/min, 95% CI [0.38, 3.99] P < 0.00001 at 24 weeks of therapy (participants in IMT: 21, participants in SC: 27, I^2^=1%). The results of the study is compared with SC after 24 weeks. The result of this meta-analysis shows that with the regular practice of IMT for 24 weeks in patients with HFpEF, the peak VO_2_ can be improved by 2.18 ml/kg/min as compared with the regular standard therapy for IMT, which includes both pharmacological and non-pharmacological therapies (Figure 4).

## Discussion

Peak VO_2_

Peak VO_2_ provides an objective assessment of cardiorespiratory fitness in patients with heart failure. It also provides prognostic significance for survival in patients with heart failure. The normal peak VO_2_ during exercise is more than 20 ml/kg/min in a healthy individual. According to a study by Mancini et al. peak VO_2 _of less than 10 ml/kg/min is associated with a significant reduction in survival in heart failure patients [13]. Improvement in peak VO_2_ co-relates with improvement in cardiac output [8,11,13]. The oxygen absorption efficiency slope, which is a good index of the cardiorespiratory functional reserve, is shown to improve significantly with a 12-week program of IMT in the study done by Stein et al. in patients with heart failure [14]. In a study by Dall’ago et al., the author found improvement in cardiorespiratory fitness as shown by a 19% increase in 6MWD and a 17% increase in peak VO_2_. His study showed a significant improvement in cardiorespiratory fitness after 12 weeks of IMT. Nonetheless, we don’t know whether these effects are transient or sustained. IMT has been shown to improve peak VO_2_ in HFrEF patients; however, there is a paucity of data to comment on the role of IMT in HFpEF [11,14]. This is further supported by the study by Fernandez-Rubio et al. highlights that IMT is effective in improving functional capacity in patients with heart failure, both with preserved and reduced ejection fractions [2].

Inspiratory muscle weakness (IMW)

Many studies link the beneficial role of IMT only in patients with IMW [12,14-15]. Even though we lack studies to show that IMW is a major pathophysiological mechanism to cause HFpEF but a study by Yamada et al. shows that IMW is associated with exercise intolerance in patients with HFpEF. Patients with IMW had a shorter 6MWD compared to patients without IMW (all P < .05). Yamada et al. commented that impaired diaphragm muscle thickening at end-inspiration (median value < 3.9 mm) was significantly associated with a high prevalence of IMW and reduced 6MWD [16]. However, the study by Palau et al. shows that irrespective of baseline IMW, IMT improves aerobic capacity, especially in older patients [12].

Duration of IMT

All four of our studies showed benefit at 12 weeks of IMT and two of our studies compared the changes in peak oxygen consumption after 24 weeks of therapy and the pooled effect showed improvement in the peak oxygen consumption as compared to SC. This answers that IMT should not be stopped at 12 weeks of therapy and continued till 24 weeks for a prolonged benefit [10-11]. 

IMT and Cardiovascular Mortality

IMT has not been proved to improve cardiovascular and all-cause mortality in patients with HFrEF and the data on the impact of IMT on HFpEF is scarce [2,11,14-15]. All these studies support the role of IMT in HFpEF. In our study, the pooled increase in Peak VO_2_ of 2.82 ml/kg/min and increase in 6MWD of 83.97 meters at 12 weeks of IMT is not only statistically significant but also clinically significant. In a study by Mancini et al., the increase in Peak VO_2_ from 10 ml/kg/min to 14 ml/kg/min in heart failure patients was associated with a high increase in cumulative survival [13]. In a study by Winkelmann et al., the addition of both IMT and aerobic exercise has shown to improve peak VO_2_ by 40% as compared to the 20% improvement with aerobic exercise alone in patients with heart failure [17]. Even though the RCT included in our meta-analysis showed a consistent benefit, we need a bigger sample size to strongly recommend IMT and prove its benefit in preventing cardiovascular mortality and increasing survival [8,10,11-12].

Summary of IMT in HFpEF

**Figure 5:**
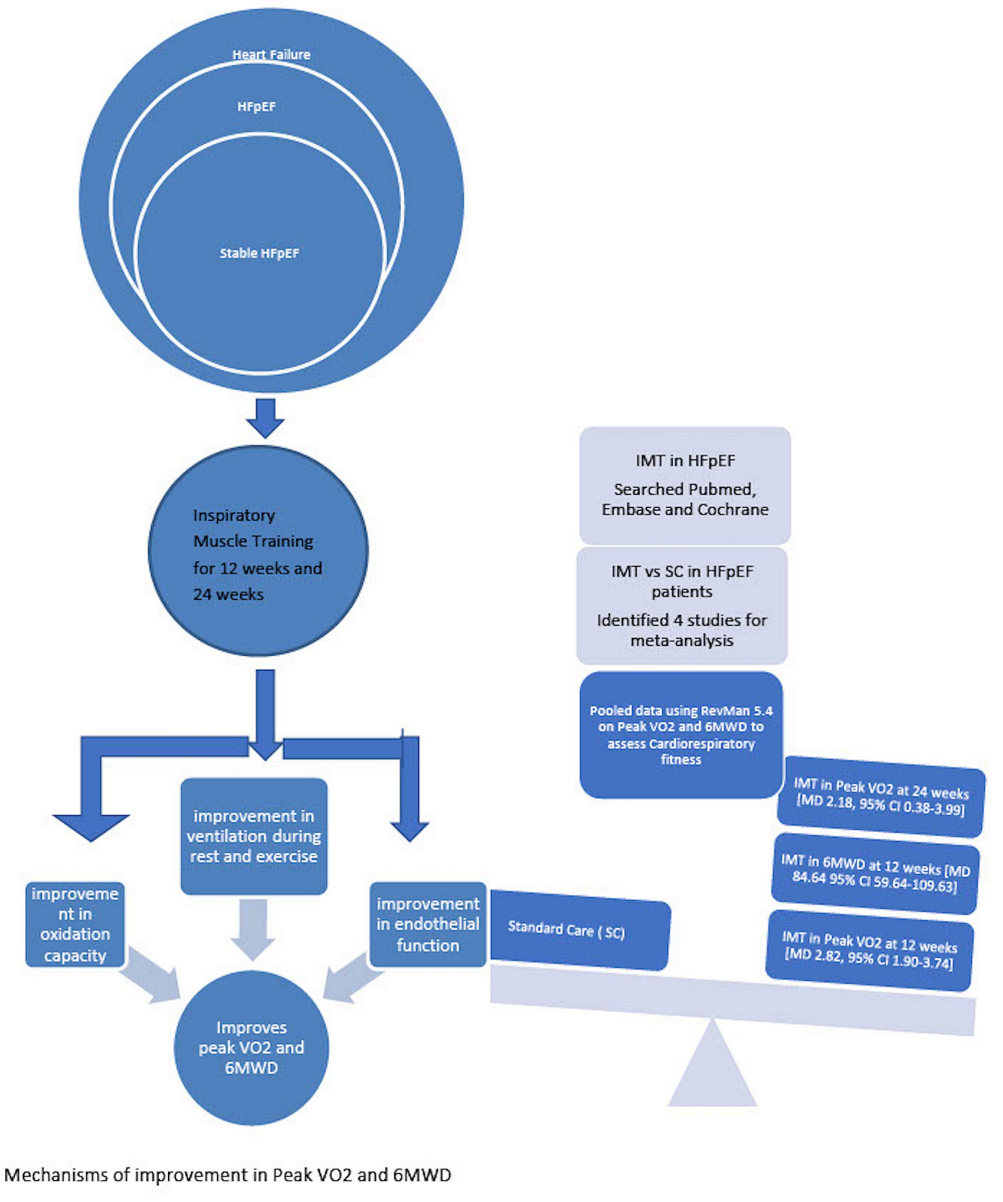
Summary of IMT in HFpEF IMT: Inspiratory Muscle Training; HFpEF: Heart Failure With Preserved Ejection Fraction

The pathogenesis of HFpEF includes excessive collagen deposition and left ventricular (LV) stiffness, endothelial dysfunction, decreased oxidation capacity, and cellular damage by inflammatory mediators [18]. By improving ventilation and oxidation capacity, endothelial function, and IMW, IMT may play a role in improving the Peak VO_2_, 6MWD, and cardiorespiratory fitness in patients with stable HFpEF. In our meta-analysis of three RCTs and one prospective study, the pooled increase in Peak VO_2_ of 2.82 ml/kg/min and increase in 6MWD of 83.97 meters at 12 weeks of IMT and increase in Peak VO_2 _of 2.18 ml/kg/ml at 24 weeks of IMT as compared to SC is not only statistically significant but also clinically significant [8,10,11,12]. In a study by Mancini et al., an increase in Peak VO_2_ from 10 ml/kg/min to 14 ml/kg/min in heart failure patients was associated with a high increase in cumulative survival as shown in Figure 5 [13].

Sensitivity analysis

It showed no change in the pooled results after excluded individual studies. An RCT on the role of yoga and breathing exercise in HFpEF is currently ongoing so we couldn’t analyze its data but once it will be complete, it will certainly be helpful to explore more on the topic of breathing [6].

Potential limitations and bias

Our study did not comment on the cardiovascular mortality and hospitalizations prevented by the practice of IMT as compared to SC. We could only comment on cardiorespiratory fitness. The use of Peak VO_2 _has its own limitations. It is affected by age, gender, and baseline exercise capacity. Our study couldn't comment on how the individual study addressed these covariates.

## Conclusions

IMT has been shown to improve diaphragmatic fatigue, ventilation, oxidative capacity, and endothelial function. The pathogenesis of HFpEF includes excessive collagen deposition and LV stiffness, endothelial dysfunction, decreased oxidation capacity, and damage by inflammatory mediators. IMT has also been shown to improve ventilation, oxidation capacity, endothelial function, and IMW. There is a paucity of data on the mechanisms by which IMT decreases systemic inflammation and brings changes in the cardiovascular system. IMT is a simple, safe, low-cost, and feasible home-based therapy that can be taught to patients or caregivers in stable HFpEF. Even though there are very few RCTs on the topic of IMT in HFpEF, all studies showed consistent improvement in the quality of life, peak VO_2_, and cardiorespiratory fitness in patients with HFpEF. We couldn’t comment on the cardiovascular morbidity, mortality, and the number of hospitalizations prevented by IMT. We need a large RCT to strongly recommend IMT and further explore it as a treatment option to prolong survival and improve cardiovascular mortality.
